# Predictors of stress in patients with Lupus

**DOI:** 10.3389/fmed.2022.986968

**Published:** 2022-09-29

**Authors:** Meenakshi Jolly, Patricia Katz

**Affiliations:** ^1^Department of Medicine, Rush University Medical Center, Chicago, IL, United States; ^2^Department of Medicine, University of California, San Francisco, San Francisco, CA, United States

**Keywords:** SLE, stress, quality of life, longitudinal analysis, biopsychosocial model

## Abstract

**Background:**

Stress is common in patients with Systemic Lupus Erythematosus (SLE), and is associated with depression, fatigue, and disease flares. Stress may be modifiable and identifying those at high risk allows clinicians and allied health care professionals to develop a multidisciplinary management plan to direct appropriate resources. This study is aimed at identifying predictors of high stress over time among patients with SLE.

**Methods:**

Longitudinal data from two interviews of the Lupus Outcomes Study 2 years apart from 726 patients with SLE were analyzed for stress, measured using the Perceived Stress Scale (PSS; high-stress PSS ≥6). *T*-test and Chi-square analyses compared patient characteristics by high-stress status. Logistic regressions were conducted with high stress as the dependent variable. Covariates included demographics, disease features, quality of life (QOL), health care utilization (HCU), and comorbidities. QoL was measured using the SF-36 form (Physical Component Score, PCS; Mental Component Score, MCS) and MOS Cognitive Functioning Scale (CFS). HCU indicated having established care with a rheumatologist, use of an emergency room or hospitalization, and quality of care. *P* ≤ 0.05 were considered significant.

**Results:**

The mean age of the cohort was 50.6 (12.5) years, 92% were women and 68% were Caucasian. The mean (SD) PSS was 5.3 (3.6), and high stress (PSS >6) was noted in 253 participants. Those with high stress were more frequently below the poverty line and less commonly employed. They had a greater prevalence of comorbidities and HCU; and worse disease severity (activity, flare, damage) and QOL. In regression analyses, high stress (baseline) was associated with younger age, married status, worse QOL, and presence of diabetes. Better QOL (PCS, MCS) independently predicted decreased odds of high stress, while high stress (baseline) predicted high stress (OR 3.16, 95% CI 1.85, 5.37, *p* < 0.0001) at follow-up, after adjusting for demographics, disease features, HCU, and comorbidities.

**Conclusion:**

Patients with SLE should be routinely screened for QOL and stress during their clinical care, to identify those at risk for poor health outcomes. This information can facilitate multidisciplinary management for those at risk for worse health outcomes.

## Introduction

Quality of life (QOL) is adversely impacted in all domains in patients with Systemic Lupus Erythematosus (SLE) (1). The cumulative impact of SLE on patients QOL may exceed that of some of the other common chronic diseases (2). Adverse effects of SLE are also evident in their relationships and the QOL of partners and informal caregivers of persons with SLE (3). Fatigue is the most prevalent symptom in SLE and is noted to be disabling by 50%. In the LUMINA study, the prevalence of fatigue exceeded 80% across ethnic groups (4), and its severity was the highest among Caucasians (5). It is commonly perceived as an area of unmet needs by patients with SLE, with 54% identifying this to be a “moderate/high need” (6). No association was reported between fatigue and disease activity or damage in a study that included patients with and without fibromyalgia (5). In a study of 116 SLE patients without fibromyalgia, we did not find any association between fatigue and disease activity (7). However, we found a significant association of fatigue with stress, depression, and pain (7), where stress had the largest contribution toward fatigue (7). Stress and depression were correlated with fatigue in an analysis from the Lupus Outcomes Study that included 678 SLE patients. Stress and depression collectively accounted for 63% of the variance in fatigue, after adjusting for age, sex, disease duration, disease activity, damage, pain, fibromyalgia, and obesity (8). Stress had a greater contribution to fatigue than depression. Stress independently predicted fatigue over time, and the effects were mediated through depression (8). Any decline in stress was predictive of a clinically significant reduction in fatigue over time (8). We have also previously noted stress to be associated with cognitive dysfunction and poor body image in SLE (9, 10).

Stress is common among patients with SLE. Almost half of patients report major life stress in the past 6 months (11). Stress contributes to flares (12, 13) and disease activity (14). Furthermore, recent research validates the role of psychosocial trauma and associated stress responses in incidents (15) and prevalent SLE (16). Causes of stress in SLE may be multifactorial (17, 18), and some are modifiable (18). Biofeedback-assisted Cognitive Behavioral Therapy, Chronic Disease Self-Management Program, and Mindfulness-Based Stress Reduction Programs have each demonstrated improvements in stress (19–21), with an effect size of 0.41–0.49 (21).

We undertook this study to evaluate correlates and predictors of high stress among patients with SLE, with the goal of identifying variables that may be used in clinical practice to identify those at high risk and directing resources to them.

## Methods

Data were obtained from the University of California, San Francisco (UCSF) Lupus Outcomes Study (LOS), a large observational cohort of patients with SLE. Enrolled patients met the 1997 American College of Rheumatology (ACR) classification criteria for SLE. Data were collected through structured telephone interviews conducted annually by trained survey workers beginning in 2002. Information on demographics, disease characteristics, medications, and healthcare use were collected. Patient-reported disease activity, damage, QOL and various other validated PROs were collected. The present study incorporated data from 726 LOS participants with data from wave 5 (the first year all relevant data elements for this analysis were collected; baseline for this analysis) and wave 7 (2-year follow-up) of the interviews. All study procedures were approved by the UCSF Committee on Human Research.

## Outcomes

The primary outcome was stress measured using the Perceived Stress Scale (PSS-4), a four-item measure that evaluates the degree of the burden regarding life demands and problems. PSS-4 Items are based on “feelings and thoughts during the last month” and responses are scored on a four-item Likert scale with 0 indicating “never” and 4 indicating “very often.” Scores range from 0 to 16, where higher scores indicate more stress. In our previous studies, PSS-4 has shown high internal consistency reliability among SLE patients, with a Cronbach's alpha of 0.97 (7).

## Statistics

All analyses were completed using SAS software. We looked at cross-sectional correlates and longitudinal predictors of stress among LOS participants. Descriptive statistics were calculated for baseline demographics, disease characteristics, and health outcomes as mean ±SD for continuous variables and frequency and proportions for categorical variables. Chi-square and Student's *t*-tests were undertaken to identify factors associated with high stress in the baseline year (wave 5). Co-variates for regression analyses were selected based on the literature review of factors known to impact stress in SLE and the above analyses. Next, we performed univariate and multivariate binary logistic regression analyses with high stress at baseline (PSS >6) as the dependent variable (wave 5). Longitudinal logistic regression analyses were performed with high stress at follow-up (wave 7) as the dependent variable.

Five sequential models were tested. Step 1 included demographic covariates [age, marital status, and socioeconomic status (SES)]. SES was represented by household income. It was chosen over other SES surrogates (e.g., currently employed, below the poverty level (household income ≤125% of the Federal poverty limit) or education status) as it correlated most of these SES surrogate variables with PSS.

For step 2, we added SLE disease variables to step 1 model. These included SLE disease activity, flare severity, damage, and use of immunosuppressive medication (yes/no). Disease activity was measured using Systemic Lupus Activity Questionnaire (SLAQ) (22), a patient-reported assessment of disease activity modeled on the physician-reported disease activity index Systemic Lupus Activity Measure (SLAM). Patient-reported numeric rating scale (NRS) of their disease activity on a scale of 0–10 was recorded, which correlated highly with SLAQ scores. As SLAQ includes the symptom of depression, which may be correlated with stress, we chose to use the NRS to denote the disease activity. SLE damage was measured using the Brief Index of Lupus Damage (BILD) (23) which is also a patient-reported measure and contains 26 of the original Systemic Lupus International Collaborating Clinics (SLICC)/American College of Rheumatology (ACR) Damage Index (SDI) items. It is scored from 0 to 31, with higher scores representing more accrued damage.

Step 3 added patient-reported QOL variables to the step 2 model. These included Physical and Mental Component Scores (PCS, MCS) derived from the Medical Outcomes Study Short Form 36 (SF-36) which is validated for use in SLE. Each score ranges from 0 to 100, with higher values representing better QOL. Cognition was measured using the self-reported MOS Cognitive Function Scale. The 6-item MOS Cognitive function scale measures six aspects of cognitive functioning, including reasoning, concentration and thinking, confusion, memory, attention, and reaction time over the past 4 weeks. Scores range from 0 to 100, where higher scores represent better functioning. The internal consistency reliability of this scale in the LOS cohort was 0.93.

For Step 4, healthcare utilization variables were added to the step 3 model. These included established care with a rheumatologist, any emergency room visits or hospitalizations in the prior year, and quality of care (QOC) (24). The latter are processing quality measures, evaluated using thirteen quality indicators amenable to self-report. QOC was estimated as the proportion of indicators met of those for which the individual was eligible.

In Step 5, comorbidities commonly seen among SLE patients were added to the step 4 model. These included the presence or absence of fibromyalgia diagnosis, hypercholesterolemia, diabetes, hypertension, cardiovascular disease (myocardial infarction or transient ischemic attack), anxiety, and depression, evaluated by the Center for Epidemiological Studies-Depression scale (CES-D).

In addition, the Step 6 model was tested for the longitudinal data that further adjusted for baseline (Wave 5) PSS levels. All *P* ≤ 0.05 were significant on two-tailed tests.

## Result

The mean age of the patients was 50.6 ± 12.6 years, and 92% of them were women (Table 1). Sixty-eight percent of patients were Caucasian, and most (85.5%) had greater than high school education. Less than half were currently employed, and 11% were below the poverty line. The most common comorbidity was hypertension (43%) followed by depression (37%).

**Table 1:** Description of the SLE cohort at baseline.

	**Mean ±SD or % (*n*)**
**Demographics**	
Age, years	50.6 ± 12.6
Women	92.1 (669)
Race/ ethnicity	
White	68.0 (494)
Hispanic	6.3 (46)
African American	8.5 (62)
Asian	17.1 (124)
Married or living with a partner	57.0 (413)
education	
>High school	85.5 (621)
Employed	45.7 (332)
Below poverty line	11.2 (80)
**Disease features**	
Disease duration	16.6 ± 8.4
Disease severity	
SLE activity (NRS)	4.2 ± 2.7
Activity score-SLAQ	12.5 ± 7.9
Flare severity	
None	52.1 (372)
Mild	17.0 (121)
Moderate	21.1 (151)
Severe	9.8 (70)
Damage score-BILD	2.2 ±2.0
**Other conditions**	
Diabetes	8.6 (62)
Hypertension	43.3 (314)
Heart disease	13.0 (93)
Myocardial infarction	1.5 (11)
Transient ischemic attack	3.3 (24)
Cancer	4.0 (29)
Depression	37.3 (270)
Anxiety	23.5 (170)
Fibromyalgia	27.2 (194)
**Medications**	
Oral steroids	40.4 (293)
Immunosuppressants	36.4 (264)
**Health care utilization**	
Has a rheumatologist	77.2 (559)
Number of rheum. visits	3.7 ± 4.1
Emergency room visit-any	39.5 (286)
Emergency room visit for SLE	20.7 (146)
Hospitalized	22.9 (166)
Quality of care	8.2 ± 2.0
**Health outcomes**	
Perceived stress scale-PSS	5.3 ± 3.6
Physical component score-PCS	37.9 ± 12.2
Mental component score-MCS	47.2 ± 12.1
MOS cognition scale	69.8 ± 22.2
Center for epidemiologic studies-depression-CESD	14.1 ± 12.4

The mean disease duration was 16.6 ± 8.4 years. The mean SLE disease activity on NRS and SLAQ was 4.2 ± 2.7 and 12.5 ± 7.9, respectively. Approximately 48% of patients reported having an SLE flare. Mean BILD damage was 2.2 ± 2.0. Forty percent of patients were taking oral corticosteroids, while 36% were on an immunosuppressive agent for their SLE. Over 75% of patients had established care with a rheumatologist. The mean number of visits to a rheumatologist was 3.7 ± 4.1 in the past year. The mean QOC indicators met were 8.2 (2.0).

The mean stress score on the PSS was 5.3 ± 3.6. The mean PCS and MCS were 37.9 ± 12.2 and 47.2 ± 12.1, respectively. Mean Cognition Function Scale and CES-D scores were 69.8 ± 22.2, and 14.1 ±12.4, respectively. High stress (PSS >6) was noted among 253 individuals. Mean PSS scores among those with high stress were 9.4 ±2.2 compared to 3.2 ± 2.0 among those without (p <0.001). High-stress participants more had income below the poverty line, were less often employed, had greater disease activity and flare severity, and damage (Table 2). Patients with high stress have a greater prevalence of all the included comorbidities (except for hypertension and cancer) and had worse scores on PCS, MCS, cognition, and depression scales. Patients with greater stress more often had rheumatologist care and visited them more often, were seen at the emergency room more often (for non-SLE or SLE causes), were hospitalized more often and had worse QOC than those without high stress. In multiple logistic regression analyses, the covariates independently associated at baseline with high stress were younger age, being married (OR 1.94, 95% CI 1.10, 3.42, p 0.02), less disease activity (OR 0.84, 95% CI 0.73, 0.98, p 0.03), worse QOL (PCS, MCS and cognitive function), and having comorbid diabetes (OR 2.6, 95% CI 1.11, 6.02, p 0.03) (Table 3).

**Table 2:** Comparison by level of stress.

**Variable**	**PSS ≤ 6**	**PSS >6**	
	**(*n* = 471)**	**(*n* = 253)**	
**Demographics**			
Age, years	50.3 ± 13.5	51.1 ± 10.8	0.40
Women	90.7 (427)	94.9 (240	0.06
Caucasian	74.3 (350)	76.7 (194)	0.53
Below poverty line	7.3 (34)	18.3 (45)	<0.0001
More than high school education	87.3 (411)	82.6 (209)	0.10
Employed	50.5 (238)	37.2 (94)	0.0006
**Disease features**			
Disease duration, years	16.8 ± 8.6	16.3 ± 8.1	0.47
SLE activity (NRS-SLAQ)	3.6 ± 2.6	5.25 ± 2.5	<0.0001
Flare severity
• None • Mild • Moderate • Severe	59.6 (276) 17.5 (81) 16.4 (76) 6.5 (30)	38.6 (96) 15.7 (39) 30.1 (75) 15.7 (39)	<0.0001
Damage- BILD	2.1 ± 1.9	2.6 ± 2.2	<0.0001
**Other conditions**			
Hypertension	42.5 (200)	44.3 (1,120)	0.69
Anxiety	12.7 (60)	43.4 (109)	<0.0001
Depression	24.3 (114)	61.5 (155)	<0.0001
Fibromyalgia	19.8 (92)	40.7 (100)	<0.001
Heart disease	10.5 (49)	17.1 (43)	0.014
Cancer	3.2 (15)	5.5 (14)	0.16
Diabetes mellitus	6.0 (28)	13.4 (34)	0.0012
Myocardial infarction	0.6 (3)	3.2 (8)	0.02
Transient ischemic accident	2.1 (10)	5.6 (14)	0.017
**Medications**			
Oral steroids	40.1 (189)	41.1 (104)	0.81
Immunosuppressive medication	34.0 (160)	40.7 (103)	0.075
**Health outcomes**			
Physical component score	40.1 ± 12.4	33.8 ± 10.7	<0.001
Mental component score	52.7 ± 8.4	36.9 ± 11.1	<0.001
Perceived stress score	3.2 ± 2.0	9.4 ± 2.2	<0.001
Center for epidemiologic studies-depression score	8.2 ± 7.5	25.1 ± 12.0	<0.001
MOS cognition scale score	77.9 ± 18.0	55.2 ± 21.5	<0.001
**Health care utilization**			
Has rheumatologist	74.7 (351)	81.8 (207)	0.033
Number of rheum visits	3.4 ± 4.22	4.1 ± 4.0	0.037
ER visits	33.3 (157)	50.8 (128)	<0.0001
ER visits, SLE-related	15.7 (72)	29.5 (73)	0.0003
ER or hospitalization	24.4 (115)	34.4 (87)	0.0054
QOC indices	8.4 ± 1.8	7.9 ± 2.3	0.0009

Tabled values are Mean ± SD or % (n).

**Table 3:** Logistic regression models for high stress (PSS > 6) at Baseline (wave 5).

**Baseline model (*****n*** = **724)**				
	**Variable**	**OR**	**95% CI**	***P*** **-value**
**Step 1**	Age	1.00	(0.99, 1.01)	0.73
	Married	1.63	(1.13, 2.36)	0.0095
	Income	0.75	(0.67, 0.83)	<0.0001
**Step 2**	Age	0.99	(0.97, 1.00)	0.10
	Married	1.89	(1.27, 2.82)	0.0017
	Income	0.80	(0.71, 0.89)	0.0001
	SLE activity	1.19	(1.09, 1.30)	0.0002
	Flare severity	1.19	(0.96, 1.47)	0.12
	Damage score	1.08	(0.99, 1.18)	0.09
	Immunosuppressive use	1.10	(0.77, 1.58)	0.59
**Step 3**	Age	0.98	(0.96, 1.00)	0.051
	Married	1.77	(1.06, 2.95)	0.03
	Income	0.89	(0.76, 1.03)	0.12
	SLE activity	0.87	(0.76, 1.00)	0.045
	Flare severity	1.20	(0.91, 1.60)	0.20
	Damage score	0.99	(0.88 1.12)	0.87
	Immunosuppressive use	1.58	(1.00, 2.50)	0.053
	Physical component score	0.96	(0.93, 0.98)	0.0007
	Mental component score	0.86	(0.84, 0.89)	<0.000
	Cognitive functioning scale	0.99	(0.97, 1.00)	0.025
**Step 4**	Age	098	(0.96, 1.00)	0.054
	Married	1.91	(1.12, 3.27)	0.018
	Income	0.89	(0.70, 1.04)	0.13
	SLE activity	0.85	(0.73, 0.98)	0.28
	Flare severity	1.28	(0.96, 1.73)	0.10
	Damage score	1.00	(0.88, 1.13)	0.96
	Immunosuppressive use	1.47	(0.87, 2.29)	0.16
	Physical component score	0.96	(0.93, 0.99)	0.003
	Mental component score	0.86	(0.84, 0.89)	<0.0001
	Cognitive functioning scale	0.98	(0.97, 1.00)	0.009
	Rheumatologist	1.69	(0.84, 3.23)	0.14
	Emergency room or hospitalization	1.11	(0.65, 1.88)	0.70
	Quality of care	0.99	(0.88, 1.12)	0.88
**Step 5**	Age	0.98	(0.96, 1.00)	0.048
	Married	1.94	(1.10, 3.42)	0.022
	Income	0.86	(0.73, 1.01)	0.07
	SLE activity	0.84	(0.73, 0.98)	0.03
	Flare severity	1.29	(0.94, 1.77)	0.11
	Damage score	0.94	(0.81, 1.09)	0.39
	Immunosuppressive use	1.60	(0.96, 2.66)	0.07
	Physical component score	0.97	(0.94, 1.00)	0.03
	Mental component score	0.86	(0.84, 0.89)	<0.0001
	Cognitive functioning scale	0.98	(0.97, 1.00)	0.015
	Rheumatologist	1.50	(0.75, 2.99)	0.25
	Emergency room or hospitalization	1.10	(0.63, 1.92)	0.74
	Quality of care	1.01	(0.89, 1.15)	0.88
	**Variable**	**OR**	**95% CI**	***P*** **-value**
	Fibromyalgia	1.45	(0.83, 2.51)	0.19
	Heart disease	1.45	(0.65, 3.21)	0.37
	Diabetes	2.58	(1.11, 6.02)	0.03
	Hypertension	0.77	(0.46, 1.29)	0.32
	Cardiovascular disease	2.25	(0.57, 8.83)	0.24
	Anxiety	1.13	(0.60, 2.12)	0.70
	Depression	1.22	(0.70, 2.11)	0.48

There were changes in stress over time (from baseline at wave 5 to follow-up at wave 7). Sixty-six percent of participants had PSS>6 at both baseline and follow-up. However, 34% of participants who had PSS>6 at follow-up did not have PSS >6 at baseline. Eighteen percent of participants who had PSS>6 at baseline did not have PSS>6 at follow-up (*p* < 0.0001).

Regression analyses of the longitudinal data revealed lower QOL (PCS, MCS), and baseline stress (PSS>6) to be independent predictors of high stress at follow up (Table 4). Patients with high stress (PSS>6) at baseline were three times more likely to have high stress (PSS>6) at follow-up.

**Table 4:** Logistic regression models for high stress (PSS>6) longitudinally (wave 7).

**Longitudinal model (*****n*** = **606)**				
	**Variable**	**OR**	**95% CI**	***P*** **-value**
**Step 1**	Age	0.99	(0.98, 1.01)	0.40
	Married	1.47	(0.94, 2.10)	0.09
	Income	0.76	(0.68, 0.86)	<0.000
**Step 2**	Age	0.98	(0.98, 1.00)	0.05
	Married	1.57	(1.03, 2.40)	0.036
	Income	0.82	(0.72, 0.93)	0.0014
	SLE activity	1.16	(1.05, 1.28)	0.0025
	Flare severity	1.13	(0.90, 1.43)	0.29
	Damage score	1.09	(0.99, 1.20)	0.07
	Immunosuppressive use	0.95	(0.64, 1.41)	0.78
**Step 3**	Age	0.98	(0.96, 1.00)	0.046
	Married	1.28	(0.80, 2.03)	0.31
	Income	0.91	(0.99, 1.05)	0.20
	SLE activity	0.91	(0.80, 1.03)	0.13
	Flare severity	1.10	(0.85, 1.43)	0.48
	Damage score	0.99	(0.89, 1.11)	0.90
	Immunosuppressive use	1.19	(0.77, 1.83)	0.45
	Physical component score	0.94	(0.92, 0.97)	<0.0001
	Mental component score	0.92	(0.90, 0.94)	<0.0001
	Cognitive functioning scale	1.00	(0.99, 1.01)	0.91
**Step 4**	Age	0.98	(0.97, 1.00)	0.10
	Married	1.25	(0.77, 2.01)	0.37
	Income	0.93	(0.81, 1.08)	0.33
	SLE activity	0.90	(0.79, 1.03)	0.13
	Flare severity	1.11	(0.85, 1.45)	0.46
	Damage score	0.99	(0.88, 1.11)	0.84
	Immunosuppressive use	1.14	(0.73, 1.79)	0.57
	Physical component score	0.94	(0.92, 0.97)	<0.0001
	Mental component score	0.92	(0.90, 0.94)	<0.0001
	Cognitive functioning scale	1.00	(0.99, 1.02)	0.56
	Rheumatologist	1.19	(0.65, 2.18)	0.57
	Emergency room or hospitalization	1.18	(0.73, 1.90)	0.51
	Quality of care	0.97	(0.88, 1.08)	0.61
**Step 5**	Age	0.99	(0.97, 1.01)	0.18
	Married	1.24	(0.75, 2.07)	0.41
	Income	0.92	(0.79, 1.07)	0.27
	SLE activity	0.88	(0.77, 1.02)	0.08
	Flare severity	1.16	(0.87, 1.54)	0.32
	Damage score	0.99	(0.86, 1.13)	0.83
	Immunosuppressive use	1.22	(0.76, 1.95)	0.42
	Physical component score	0.95	(0.92, 0.98)	0.0003
	Mental component score	0.92	(0.92, 0.95)	<0.0001
	Cognitive functioning scale	1.01	(0.99, 1.02)	0.47
	Rheumatologist	1.15	(0.62, 2.16)	0.65
	Emergency room or hospitalization	1.21	(0.73, 2.02)	0.46
	Quality of care	1.00	(0.90, 1.12)	0.98
	**Variable**	**OR**	**95% CI**	***P*** **-value**
	Fibromyalgia	1.52	(0.93, 2.48)	0.09
	Heart disease	1.10	(0.52, 2.31)	0.81
	Diabetes	2.08	(0.98, 4.41)	0.06
	Hypertension	0.62	(0.39, 1.00)	0.51
	Cancer	1.06	(0.31, 3.66)	0.92
	Anxiety	1.49	(0.86, 2.61)	0.16
	Depression	1.17	(0.71, 1.94)	0.53
**Step 6**	Age	0.99	(0.97, 1.01)	0.30
	Married	1.12	(0.66, 1.90)	0.67
	Income	0.93	(0.79, 1.09)	0.35
	SLE activity	0.90	(0.78, 1.04)	0.17
	Flare severity	1.10	(0.82, 1.48)	0.53
	Damage score	1.00	(0.87, 1.14)	0.96
	Immunosuppressive use	1.17	(0.72, 1.90)	0.53
	Physical component score	0.95	(0.93, 0.98)	0.0007
	Mental component score	0.94	(0.92, 0.97)	0.0001
	Cognitive functioning scale	1.01	(1.00, 1.03)	0.21
	Rheumatologist	1.12	(0.59, 2.10)	0.74
	Emergency room or hospitalization	1.23	(0.73, 2.08)	0.43
	Quality of care	1.00	(0.89, 1.12)	0.97
	Fibromyalgia	1.43	(0.86, 1.12)	0.17
	Heart disease	1.09	(0.51, 2.32)	0.83
	Diabetes	1.73	(0.80, 3.76)	0.17
	Hypertension	0.64	(0.39, 1.04)	0.07
	Cancer	0.91	(0.27, 3.12)	0.89
	Anxiety	1.45	(0.82, 2.58)	0.20
	Depression	1.14	(0.68, 1.91)	0.62
	Stress PSS >6 baseline	3.16	(1.85, 5.37)	<0.0001

## Discussion

The impact of SLE or its treatment on a patient's psycho-social functioning and QOL may lead to implications for long-term health behaviors and downstream health outcomes. Accepting the disease or the need for ongoing medical care, medications with adverse side effect profile, impact on personal (including relationships) and vocational growth, independence, the strain on financial and internal resources, changes in appearance or function, the unpredictability of flares, pain, poor sleep, loss of control on self, may all contribute to stress. We know the importance of the mind-body connection in health and that stress can result in disease flares in SLE (12, 13, 17, 25, 26). Alexithymia has been noted in patients with SLE (27). Concerns about reproductive and sexual health (28, 29) from the disease or its medications may add to the stress. Negative effects of SLE on relationships and the health of their informal caregivers have been reported (3, 30). Stress along with the number of symptoms, anxiety, and depression were correlated with QOL in SLE (31). As noted earlier, stress is an independent and largest contributing predictor of fatigue in SLE, the effects of which are mediated through depression (8). In our previous analyses, any reduction in stress is associated with a large and significant reduction in fatigue (8). If stress can be improved, patients with SLE may be able to cope better with the disease, flares, their impact on their daily lives, their medical care, and possibly resultant urgent and emergent health care utilization.

In our study, SLE patients with high stress had low incomes and were less likely to be employed. Correlations of stress with education, income, poverty, and number of symptoms have been previously reported (17, 31). We did not find an association between stress and education. Part of the reason for the lack of association with education may have been a lack of variability in educational status, as over 80% of patients had more than a high school education. Younger participants had higher stress, and this could potentially be from the challenges imposed by the chronic disease diagnosis at a young age, the need for frequent and long-term management, adverse effects on relationships, procreation, vocational training, and limited internal or external resources (including financial independence or reserves to cope with the disease or its management). Surprisingly, we noted in our study that being married is associated with greater stress. It is known that less stress and better mental health are seen among married people than single (32, 33), especially among men (34). It is plausible that we noted greater stress among married participants in our study first because majority of participants were women, and second SLE impacts not only patients' health and daily life but also the quality of their relationship and intimacy with their partner (3), procreation, and vocation.

Unlike other studies (17, 31), lower disease activity was correlated with higher stress. We have previously reported greater disease activity to be associated with higher patient satisfaction with care (35). This might reflect patients' expectations anchored around acute illness or a result of greater interactions with the health care systems and providers. The presence of most comorbidities as expected was associated with greater stress but the use of steroids or immunosuppressive medications was not. In the multivariate models, only diabetes out of other comorbidities was independently associated with high stress (Table 3). Lack of association with immunosuppressive medications could potentially be because the use of these medications may have been confounded with either disease activity or health care use. We found patients with high stress to have worse QOL (PCS, MCS), depression (CES-D), and cognitive functioning (MOS cognitive function scale). The association of stress with QOL, depression, and cognition is known (7–10, 31). Cognitive deficits may occur in SLE as part of neuropsychiatric involvement, effects of dealing with a chronic disease, comorbidities, and side effects of medications. SLE patients have shown poor decision-making, lower cognitive flexibility, and greater vulnerability to stress, some of which may be attributable to the use of corticosteroids (36). Patients with high stress in our study had greater access to rheumatologist care and had greater health care utilization indicated by a greater number of visits annually to the rheumatologist, or to the emergency room and hospitalization. This is plausible as patients with high stress had greater disease activity, the severity of flares, damage, and comorbidities. Quality of care indices met was significantly higher among those with less stress (PSS<6).

Longitudinal data models showed worse QOL (PCS, MCS) and having high stress at baseline to be the independent predictors for high stress. Being in a high-stress state at baseline had the biggest impact (OR 3.2, 95% CI 1.9, 5.4, *p* < 0.0001) on having high stress over time in our study. Stress has been found to be modifiable in some studies. Biofeedback-assisted Cognitive Behavioral Therapy, Chronic Disease Self-Management Programs, mindfulness-based stress reduction programs may lead to improvements in stress (19–21, 37, 38), with moderate to large effect sizes (21, 37). However, in a randomized clinical trial involving brief supportive-expressive psychotherapy of 133 women with SLE across 9 clinics in Canada, no improvements in psychological distress, stress, or coping were noted among the intervention and control group (39). Another study with an aerobic exercise program intervention did not result in a reduction in psychological stress in SLE (40).

There are several limitations to this study. First, the cohort was predominantly Caucasian. We did not have physician assessed disease activity and damage measures, although SLAQ and BILD are both validated measures for activity and damage and are widely used in research. Most patients had an education level of high school and greater, which may not be generalizable to other groups. We did not have detailed information on various immunosuppressive medications. Multiple comparisons in bivariate analyses were undertaken without applying Bonferroni correction. The study also has some strengths. This is the first study evaluating stress as the primary outcome in a longitudinal setting with a large cohort of patients with SLE, which includes a large number of relevant covariates. The relevant findings of QOL and high stress being independent predictors were observed after accounting for a wide variety of pertinent variables. Applying Bonferroni correction to the longitudinal analysis model 6, QOL (PCS, MCS) and baseline high stress were independent and significant predictors of high stress at follow-up.

The importance of evaluating and addressing stress in SLE patients cannot be over-emphasized. More studies are needed addressing interventions to modify stress in SLE and to systematically evaluate the downstream effects on health outcomes and health care utilization. A care coordination model such as ambulatory integration of the medical and social (AIMS) model (41) could be embedded using the Biopsychosocial model for chronic disease care for SLE patients. In conclusion, SLE patients should be routinely screened for QOL and Stress during clinical care, to identify those at risk for poor health outcomes and to facilitate multidisciplinary management plans.

## Data availability statement

The data analyzed in this study is subject to the following licenses/restrictions: Permission from UCSF is needed for use of any data related to Lupus Outcomes Study. Requests to access these datasets should be directed to Lupus Outcomes Study Patti.Katz@ucsf.edu.

## Ethics statement

The studies involving human participants were reviewed and approved by UCSF. The patients/participants provided their written informed consent to participate in this study.

## Author contributions

All authors listed have made a substantial, direct, and intellectual contribution to the work and approved it for publication.

## Funding

The Lupus Outcomes Study was funded by NIH grant P60 AR053308.

## Conflict of interest

The authors declare that the research was conducted in the absence of any commercial or financial relationships that could be construed as a potential conflict of interest.

## Publisher's note

All claims expressed in this article are solely those of the authors and do not necessarily represent those of their affiliated organizations, or those of the publisher, the editors and the reviewers. Any product that may be evaluated in this article, or claim that may be made by its manufacturer, is not guaranteed or endorsed by the publisher.
